# Growth Hormone Receptor Regulation in Cancer and Chronic Diseases

**DOI:** 10.3389/fendo.2020.597573

**Published:** 2020-11-18

**Authors:** Ger J. Strous, Ana Da Silva Almeida, Joyce Putters, Julia Schantl, Magdalena Sedek, Johan A. Slotman, Tobias Nespital, Gerco C. Hassink, Jan A. Mol

**Affiliations:** ^1^ Department of Cell Biology, Centre for Molecular Medicine, University Medical Centre Utrecht, Utrecht, Netherlands; ^2^ BIMINI Biotech B.V., Leiden, Netherlands; ^3^ Department of Clinical Sciences of Companion Animals, Faculty of Veterinary Medicine, Utrecht University, Utrecht, Netherlands

**Keywords:** aging, cancer, ubiquitin, endocytosis, GH/IGF-1 axis, GH sensitivity

## Abstract

The GHR signaling pathway plays important roles in growth, metabolism, cell cycle control, immunity, homeostatic processes, and chemoresistance *via* both the JAK/STAT and the SRC pathways. Dysregulation of GHR signaling is associated with various diseases and chronic conditions such as acromegaly, cancer, aging, metabolic disease, fibroses, inflammation and autoimmunity. Numerous studies entailing the GHR signaling pathway have been conducted for various cancers. Diverse factors mediate the up- or down-regulation of GHR signaling through post-translational modifications. Of the numerous modifications, ubiquitination and deubiquitination are prominent events. Ubiquitination by E3 ligase attaches ubiquitins to target proteins and induces proteasomal degradation or starts the sequence of events that leads to endocytosis and lysosomal degradation. In this review, we discuss the role of first line effectors that act directly on the GHR at the cell surface including ADAM17, JAK2, SRC family member Lyn, Ubc13/CHIP, proteasome, βTrCP, CK2, STAT5b, and SOCS2. Activity of all, except JAK2, Lyn and STAT5b, counteract GHR signaling. Loss of their function increases the GH-induced signaling in favor of aging and certain chronic diseases, exemplified by increased lung cancer risk in case of a mutation in the SOCS2-GHR interaction site. Insight in their roles in GHR signaling can be applied for cancer and other therapeutic strategies.

## Introduction

In 1989 with a background of posttranslational modifications and intracellular transport of membrane glycoproteins our lab decided to focus on studying the role of ubiquitination in membrane trafficking. Knowledge on the role of ubiquitination as a major regulator of cell functions had just started to emerge (1). To address the question of whether ubiquitination and membrane trafficking are connected processes, we sought a model membrane protein to focus on. Some evidence suggested that the growth hormone receptor (GHR), isolated from rabbit liver, is ubiquitinated (2). We choose this as our model and very soon, it became clear that the two fields were indeed connected (3). Now, we know that both the ubiquitin system and the GHR are crucially important for the regulation of cellular life and metabolism. The state-of-the-art of both fields has been described in excellent recent reviews (4–11). In this review we will connect both systems.

Loss of the GHR is not lethal, but results in sub-optimal health, short stature, decreased bone mineral density, decreased muscle strength, thin skin and hair, increased adiposity, and hepatic steatosis. Interestingly, people with non-functional GH signaling have very low plasma insulin growth factor 1 (IGF-1) concentrations, are highly resistant to cancer and diabetes type 2 and seem to have a slow cognitive decline (12, 13). GHR, whose function is more a modulator of cellular processes, may deteriorate healthy aging and act as an important stimulator of carcinogenesis. Our focus will therefore be on the mechanisms involved in the regulation of this important receptor, wherein ubiquitination and phosphorylation enzymes play major parts, and on the impact of these in health and disease.

## The Growth Hormone Receptor

### The Prototype Cytokine Receptor

GHR is a single membrane spanning protein of 638 amino acids, isolated for the first time from rabbit liver (14). Cloning from several species revealed a strong sequence homology (15). The human *GHR* is composed by 9 exons (16) encoding a cleavable amino acid signal peptide of 18 (exon 2), an extracellular domain of 246 (exon 3 to 7), a transmembrane domain of 24 (exon 8), and an intracellular domain of 350 residues (exon 9 and 10). GHR belongs to the class 1 superfamily of cytokine receptors, which includes 27 ligands and 34 human type I cytokine receptors (17). The GHR was the first member of the family to be characterized (2) and is expressed in most cells of the human body.

**Figure 1:**
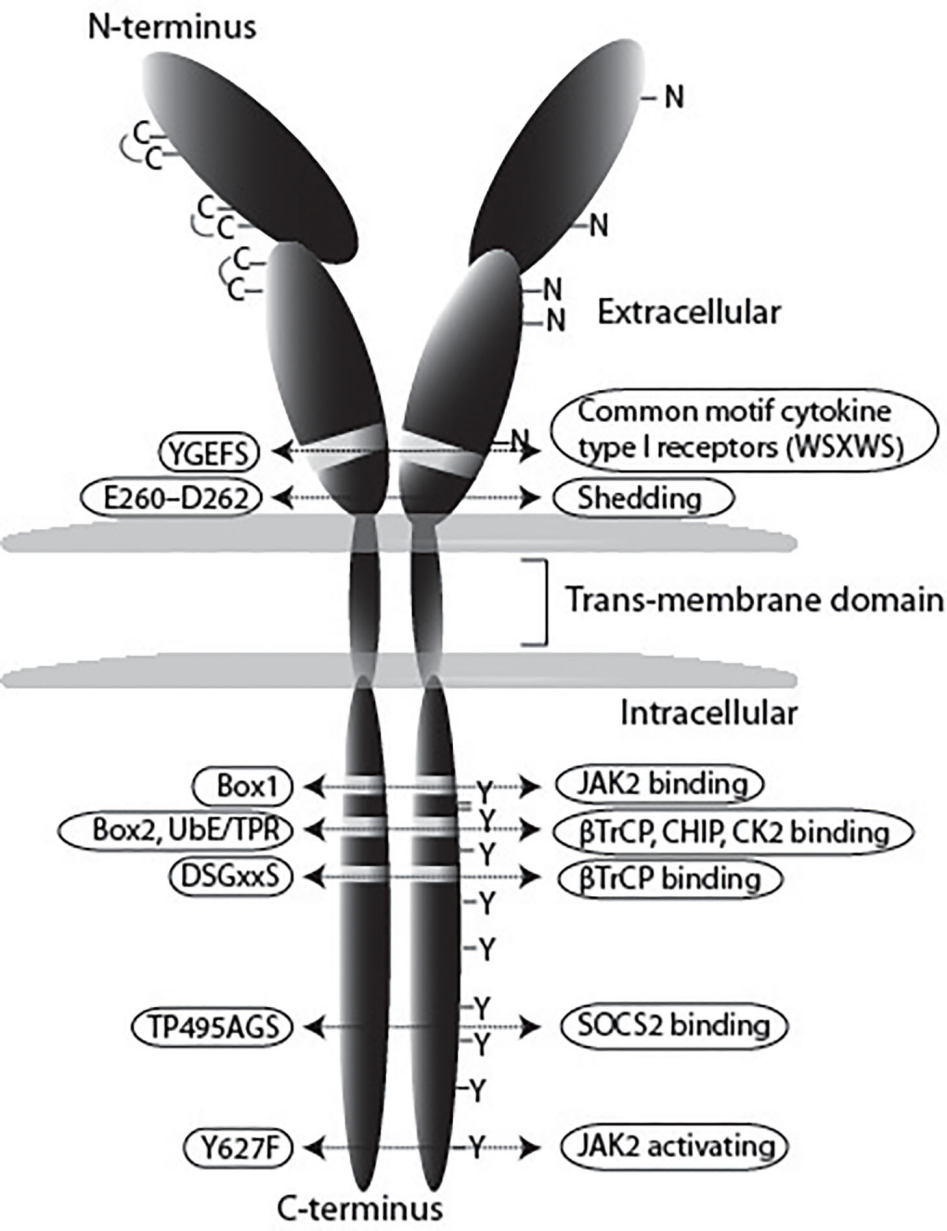
Schematic representation of the GHR. In this review we use the notation of the human GHR protein (GenBank: AAA52555). The GHR consists of an extracellular domain of 246 amino acids, a transmembrane domain (TMD) of 24 residues, and a cytoplasmic region of 350 amino acids. The extracellular domain contains 5 potential glycosylation sites (N), and seven cysteine residues, from which 6 form disulfide bonds. The YGEFS domain is located at the C-terminal part of the extracellular domain. The intracellular domain contains 9 tyrosine residues that can be phosphorylated upon receptor activation by GH. E260-D262 are involved in ADAM17 shedding activity, Box1 is responsible for JAK2 binding, the membrane-proximal 150 amino acids contain the SRC (Lyn) binding site; UbE/TPR and DSGxxS are for GHR internalization and degradation, TP495AG serves as SOCS2 binding site (21), and Y627F causes constitutive JAK2 activation (22).

The class 1 cytokine receptors share many features. In the extracellular domain they contain conserved cysteine residues and a WSxWS motif (18). In the case of GHR, this motif is different, although homologous, YGEFS. Alteration of the sequence disrupts ligand binding and receptor signaling (19). Despite the limited amino acid homology, the structures of GHR, EPOR and PRLR are similar, consisting of two fibronectin- (FN) type 3 domains (β-sandwich composed of seven β strands). In GHR the N-terminal domain is composed of amino acids 19–141 and the C-terminal composed of amino acids 146–264, separated by a four-amino acid hinge region (20). The GHR extracellular domain contains 3 disulfide bridges, formed by 6 of its 7 cysteine residues (**Figure 1**) (23). The intracellular domain contains two conserved membrane-proximal conserved sequences, referred to as box1 and box2, equivalent to the UbE/TPR motif, with functions in JAK2 binding and GHR endocytosis, respectively. Additionally, a conserved DSGxxS degradation motif is present downstream of box2, whose function is explained later in this review.

### GHR Life Cycle

**Figure 2:**
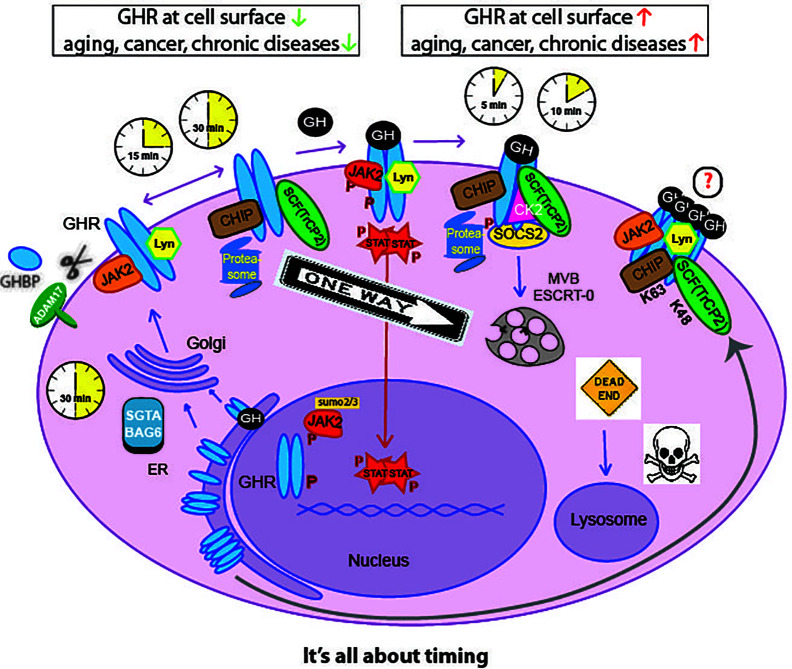
Life cycle of the GHR. GHR is synthesized in the endoplasmic-reticulum (ER) where it undergoes N-glycosylation and dimerization. The small glutamine-rich tetratricopeptide repeat-containing protein SGTA together with Large proline-rich protein BAG6 (or BCL2-associated athanogene 6), SGTA/BAG6, probably act as chaperone in GHR complex formation toward the Golgi complex. After complex glycosylation in the Golgi the GHR arrives at the plasma membrane within 30 min. With no GH present, the GHR endocytoses *via* coated pits catalyzed by βTrCP (on DSGxxS motif, **Figure 1**), Ubc13/CHIP, and the proteasome, within 30 min after arrival. Alternatively, the GH binding domain (GHBP) is shedded into the blood by the action of ADAM17. If GH binds to the GHR, signal transduction is initiated *via* JAK2, Lyn, and CK2; in addition, SOCS2 is recruited to the degron sequence TP^495^AGS, downstream of the STAT5b-interacting pY487. The activated GHR uses the same ubiquitination machinery but a different motif (UbE/TPR) for βTrCP binding and endocytosis. This shortens the residence time to 5-10 min, as explained below and in **Figure 5**. The endocytosed GHRs are then transported through endosomes, selected by the ESCRT-0 complexes at the multivesicular bodies (MVB) and degraded in the lysosomes. The CK2 involvement is hypothetical. Upon GH binding, JAK2 and Lyn phosphorylate downstream effectors. Phosphorylated STATs translocate to the nucleus and activate many genes. Both GHRs and JAK2 can be translocated to the nucleus (24–26). High-order functional GH-GHR complexes may occur that upon activation are phosphorylated and act as signaling platforms (27, 28). If GH and GHR are expressed in the same cell, they bind in the ER and the signaling starts from the Golgi complex.

While being translated on ribosomes, GHR is inserted in the endoplasmic reticulum (ER) membrane due the presence of the signal peptide (**Figure 2**). In the ER, the disulfide bonds are formed and GHR dimerizes (29). GHR is glycosylated with high mannose oligosaccharides important for the process of quality control in the ER. When correctly folded, higher order (presumably tetrameric) complexes assemble, and GHR continues its route in to the Golgi apparatus (27). In the Golgi, the high mannose oligosaccharides of GHR are processed into complex oligosaccharides.

In 2003, we identified both SGTA and BAG6 as binding partners for the GHR (30, 31). The binding depends on an intact UbE/TPR motif, similar as for CHIP and βTrCP. Also pentatricopeptide motif-containing proteins like LRP130 were identified (30). The binding was lost if F345 was mutated. Bag6/Bat3 localized to the nucleus, the Golgi complex and to mitochondria. Inhibition of protein synthesis as well as UV-treatment resulted in a reduction of Bag6 to mitochondria. According to current insight Bag6 can bind to both precursor and mature GHR *via* SGTA and Ubl4a (32–34). Silencing of UBL4 had no effect on GHR function at the cell surface and its endocytosis (35). Most likely the SGTA/Bag6 complex plays a role in GHR dimerization, and multimerization at the endoplasmic reticulum and in the Golgi complex (27, 36).

After this step, the GHRs traffic to the cell surface (**Figure 2**). Unlike most growth factor receptors, the GHR is continuously synthesized and degraded with a half-life of 30-60 min (37–39). GHR is constitutively endocytosed independently of GH binding (39, 40). GH binding at the cell surface initiates signaling, and accelerates endocytosis of the GH-GHR complex (41, 42). GHR is sorted into the multivesicular bodies (MVB), and eventually degraded in the lysosome (43). Additionally, when at the cell surface, GHRs can be cleaved by the metalloprotease tumor necrosis factor-α-converting enzyme (TACE, ADAM17), a process called shedding (44, 45). The cleaved extracellular domain circulates in the blood and is referred to as growth hormone binding protein (GHBP); the intracellular part is endocytosed and degraded. GHBP levels in the blood have been used as an indication of the amounts of GHR in the cells (46). When GH is bound to GHR or if a tri-peptide (E260–D262) is deleted or mutated, shedding is inhibited (39, 47, 48). In the bloodstream, GHBP may antagonize GH actions by competing for its binding with GHR at the cell surface (49). Alternatively, the GH-GHBP complex may increase the bioavailability of GH in the circulation. Another function of the shedding process is downregulation of the responsiveness of the cells to GH. The availability for GH at the cells surface is determined by the rate of GHR endocytosis (75%), the rate of shedding (10%), and other (unknown) mechanisms (15%) (50). Control of GHR endocytosis is crucially important. The high turnover rate allows cells to quickly respond to stresses and changing metabolic conditions.

Both GHR and its kinase, JAK2, can translocate to the nucleus. The JAK2 transport can be facilitated by the sumoylation machinery (24).Under certain conditions at defined cell cycle- regulated times in proliferative cells, activated GHR escapes *via* the cytoplasm to the nucleus by the importin- α/β mediated classical import pathway. This process requires interaction with the nuclear localization signal-containing protein Co-activator activator (CoAA). Through its N-terminal domain nuclear GHR can act as a transcriptional activator in conjunction with CoAA to initiate transcription of a subset of target genes to regulate cell cycle progression. Most likely, the nuclear GHR together with CoAA increases the proliferative action of GH (25, 26). Details as whether dimeric, phosphorylated GHR, or whether JAK2, or GH are needed, are currently lacking.

## Growth Hormone Physiology

### GH Family and Structure

Growth Hormone (GH), also known as somatotropin or somatotropic hormone, is a peptide hormone produced in the anterior pituitary gland which promotes cell division, regeneration and growth (6). Phylogenetically, GH is an ancestral hormone that has been found in the pituitary of primitive vertebrates, such as the jawless sea lamprey fish (51). In primates, GH is part of a family of highly similar genes consisting of GH1 which is mainly expressed in the pituitary, a placental GH variant gene known as GH2, and three placental lactogens also known as chorionic somatomammotropin genes (CSH1 and CSH2) and chorionic somatomammotropin-like gene (CSHL1). Several GH isoforms have been identified, but in humans the majority of the circulating GH is the 22,000 GH1 form from the pituitary gland (52, 53). Some extra-pituitary tissues (e.g. neural, immune, reproductive, digestive, respiratory systems among others) have also been found to produce GH (54). Thus, GH and also PRL expression is widely spread in many tissues throughout the body where it has autocrine or paracrine functions and may play a role in various diseases. Autocrine GH may have an even greater role in cancer development than endocrine GH (55). In mammary tissue GH1 expression has been found to be regulated by progesterone (56–58). GH belongs to the same family as prolactin (59), and in primates GH binds also to the PRLR, which presumably implicates all PRLR-mediated actions including mammary gland differentiation and lactation. Two disulfide bounds are necessary for its biological activity (60). Before closure of the growth plates, recombinant GH can be given to promote growth in children with short statue (61).

### GH Secretion and Availability

**Figure 3:**
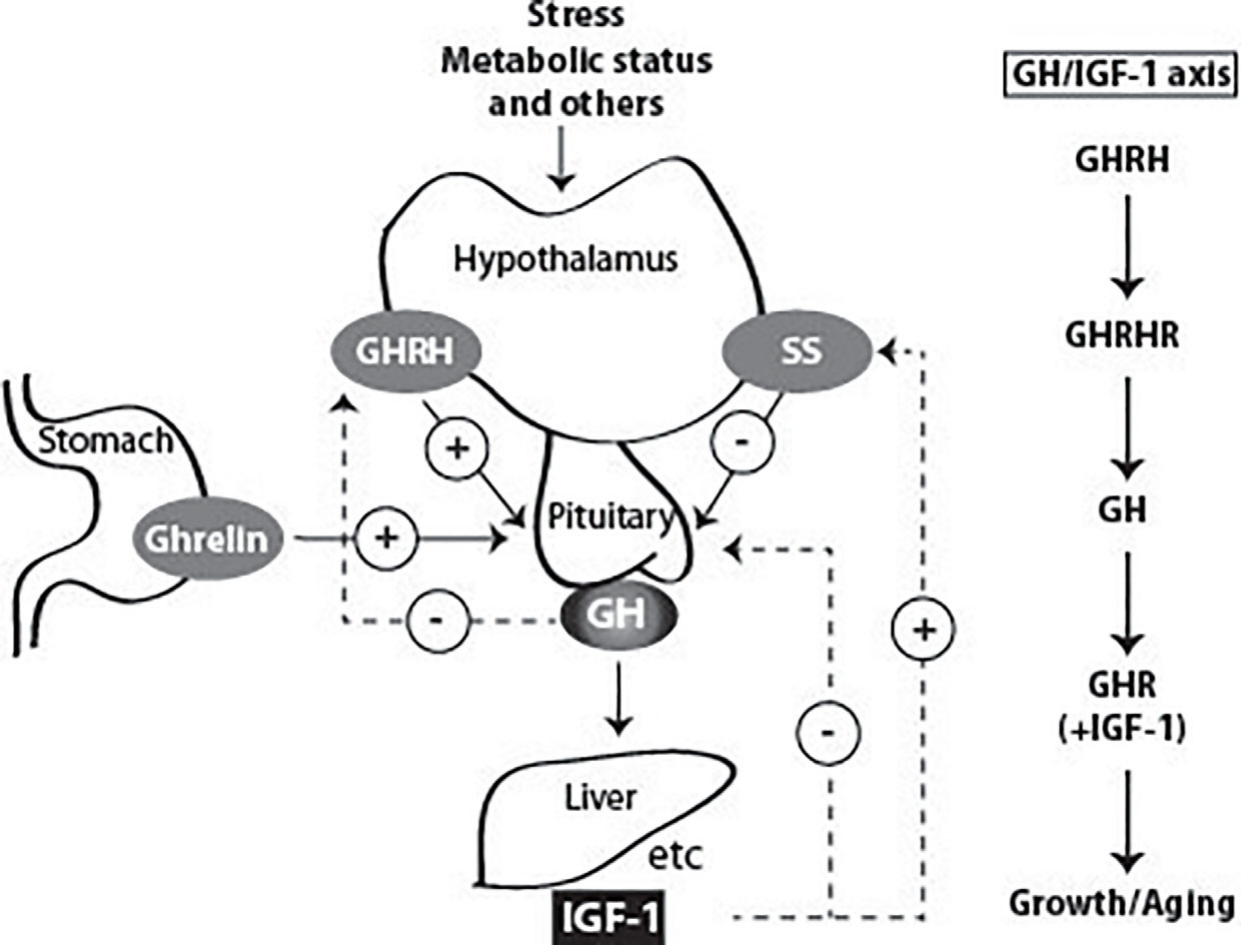
Factors that stimulate and suppress GH secretion under physiological conditions. Several factors influence GH secretion including stress, nutrition, and exercise among others. However, two factors are the main regulators: GH releasing Hormone (GHRH) and somatostatin (SS), which stimulate and inhibit GH secretion, respectively. Ghrelin produced in the stomach also stimulates GH release. GH stimulates the synthesis of IGF-1 by the liver, and in other peripheral tissues. Both GH and IGF-1 are involved in negative feedback loops. High GH levels inhibit its own secretion by inhibiting the release of GHRH. High blood levels of IGF-1 lead to decreased secretion of GH by direct suppression in the pituitary and by stimulating the release of SS.

GH is released from the somatotropic cells in the anterior pituitary in a pulsatile manner (**Figure 3**). In man, GH is secreted episodically with a major surge at the onset of the slow-wave sleep, and less pronounced secretory episodes a few hours after meals (62–64). The pulsatility of GH secretion has a major impact on the pattern of GH-induced hepatic gene expression (65–67). Sexually-dimorphic patterns of genes manifest themselves in the liver through the pulsatile nuclear and DNA occupancy of STAT5b in males, while In females a more continuous pattern leads to dramatic differences in gene expression (68–70). Pulses are regulated primarily by the interplay of hypothalamic hormones: a stimulatory GH-releasing hormone (GHRH) and an inhibitory hormone, somatostatin (SS). These factors act *via* their respective receptors, expressed at the cell surface of the somatotropic cells. In addition, other peptides, called secretagogues (GHS), were identified to regulate GH secretion, such as GH releasing peptides (GHRP) originating from the brain (71), and Ghrelin, produced by stomach tissue (72). Additionally, insulin-like growth factors (IGFs), of which the transcription depends on GH/STAT5b signaling, are able to inhibit GH release in a negative feedback loop (73). Expression and release of GH are mainly regulated by the transcription factor Pit-1, which has additional functions in the differentiation and maintenance of somatotropic cells (74, 75). GH secretion is also affected by other factors such as physical stress, body composition, metabolic status and others (**Figure 3**). For instance, during fasting and certain conditions of physical stress, GH secretion is increased, and excess of glucose or lipid intermediates inhibits GH release in healthy man (63, 76–78). After maximal GH secretion at puberty (79), adulthood is associated with its gradual decline (80). Besides the tight regulation of GH secretion, the availability of GH is also influenced by its clearance by the kidneys and by internalization through its receptor. During pregnancy, the pulsatile GH secretion is completely abolished due to the placental GH2 secretion which evokes elevated plasma IGF-1 concentrations inhibiting pituitary GH1 release by feedback inhibition. Because of this, insulin-resistance may develop eventually leading to pregnancy diabetes (81).

## GHR Activation, Signaling, and Desensitization

GH signaling not only depends on the amounts of GH in circulation, but also on the levels of GHR at the cell surface. The responsiveness (sensitivity) of the cells to GH is dynamically regulated, reflecting a balance of receptor endocytosis/degradation, and transport of newly synthesized receptors to the plasma membrane (50).

If GH and GHR are synthesized in the same cell, autocrine signaling occurs. Binding and complex formation takes place in the ER, but signaling starts only in the Golgi complex (27, 82). As it is a continuous process, the kinetics of downstream signaling certainly differ from the endocrine mode. While for the latter, JAK2-induced phosphorylation is rapidly counteracted by SOCS activity and endocytosis, the autocrine signaling occurs continuously from inside and there is no information about the exact role of the different factors discussed in this review. Most likely, cells that synthesize GH, already carry GH-GHR complexes at the cell surface and react differently upon GH from outside the cell.

### Receptor Activation Mechanisms

The class 1 cytokine receptors do not have intrinsic kinase activity (83). This role is mediated by JAK2 and the SRC kinase family member, Lyn, that associate with sequences in the cytosolic tail: box1 for JAK2 (84), and the membrane proximal 150 residues of the cytoplasmic domain for Lyn (85). In this review we choose Lyn as a member of the SRC family, but also c-Src and Fyn may be involved (86).

**Figure 4:**
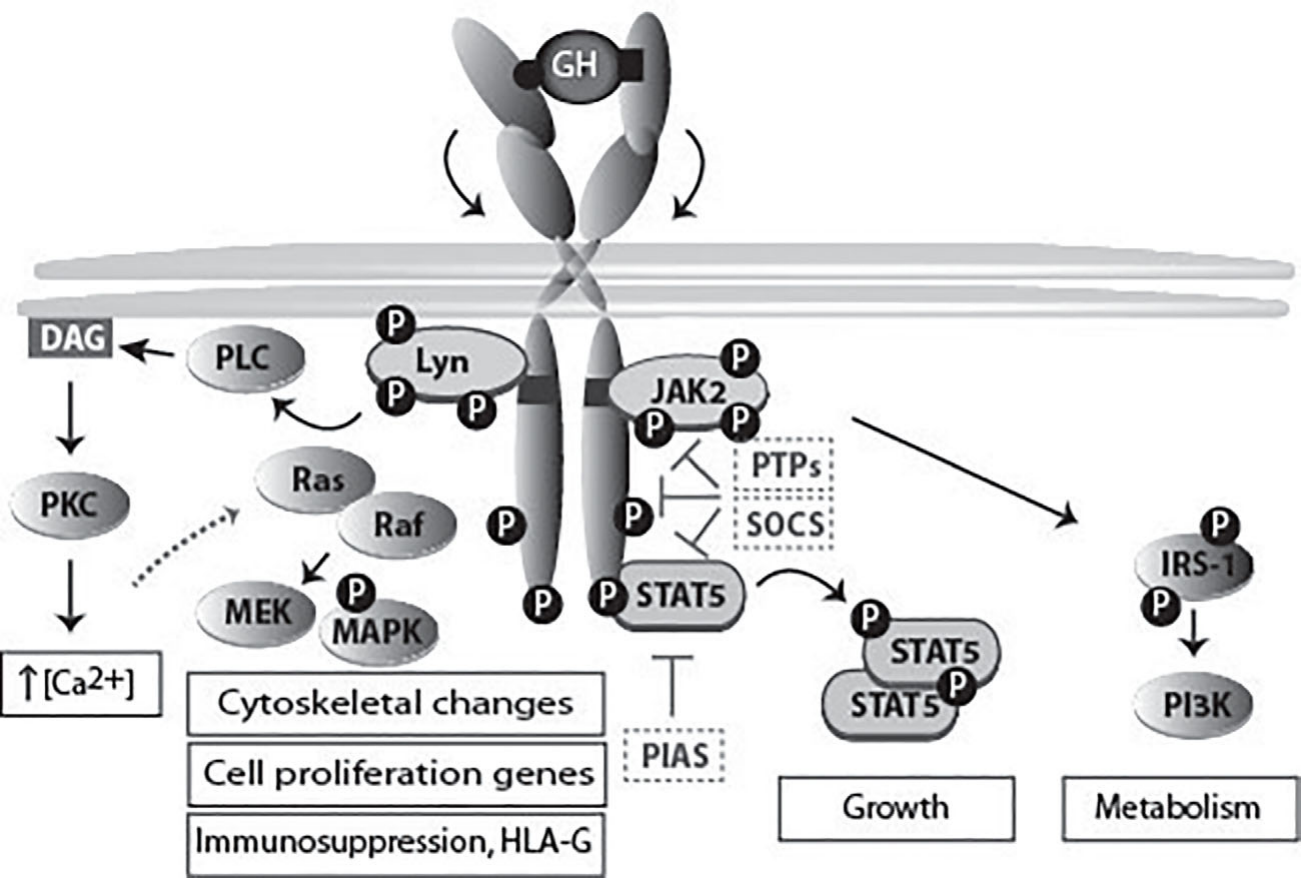
GHR activation and signaling. The binding of the two asymmetric binding sites of one GH molecule to the GHR dimer causes its rotation and subsequent activation of downstream signaling pathways, and ultimately specific gene transcription. The activation of different signaling pathways accounts for the multitude of GH functions. JAK2 binds to box1, while the SRC family member (Lyn) binding is to the membrane proximal 150 residues (85). Lyn activates PLCγ, which leads to an increase in the cytoplasmic calcium ion concentration. This process results in RAS activation and initiation of the ERK1/2 signaling pathway. In the scheme also molecules involved in signal termination (PTPs, SOCS and PIAS) are indicated. In **Figure 7** the physiology and pathophysiology are further detailed.

The first step in GH action is its binding to the GHR. The crystal structure of the extracellular domain of GHR bound to GH revealed that one GH molecule binds with two asymmetric binding sites two molecules of GHR (20) (**Figure 4**). For a long time, it was thought that GH binding to one GHR monomer at the plasma membrane recruits the second monomer of GHR to its second binding site. JAK2 activation was proposed to occur due to GHR dimerization itself. However, subsequent studies disproved this model. Studies by Gent and collaborators showed that GHR dimerizes in the ER, independently of GH, and travels to the cell surface as a pre-formed dimer (29). Subsequently, work by the group of Waters suggested that a change in conformation, induced by GH, rather than GHR dimerization, is responsible for GHR activation. In this study, the comparison of the crystal structure of the extracellular domain of GHR alone and the previous structure of GH-bound GHR revealed differences in conformation (87). Based on this knowledge, the current model for GHR activation proposes that GH binding to the GHR dimer causes a change in conformation in the extracellular binding domain This structural change causes the receptor transmembrane domain to change from a parallel to a left-handed crossover interaction. This structural transition leads to a separation of the intracellular domain, at least to the Box1–Box2 motif, dissociates the JAK2 kinase/pseudokinase trans-interaction and brings the JAK2 kinase domains in proximity, allowing trans-phosphorylation and activation (88, 89). As described for many cytokine receptors, high-order functional GH-GHR complexes of 900,000 Mr occur that upon activation are phosphorylated and act as signaling platforms as identified by native polyacrylamide gel electrophoresis (27, 28, 90).

The phosphorylation of specific tyrosine residues, brought about by JAK2 and Lyn, has extensively been studied as intermediaries for recruiting downstream signaling effectors (91). Herewith in agreement, we confirmed that tyrosine residues 487, 534, and 627, but not residue 566, are most important for GHR and STAT5b phosphorylation. In addition, we showed that the GHR(Y627F) mutation constitutively (independent of GH binding) activates JAK2 and downstream effectors (92).

### JAK2 Activation

JAK2 is composed of four major domains: a N-terminal FERM (4.1 protein, ezrin, radixin, and moesin) domain, followed by a SH2 (Src homology 2) domain, pseudokinase and kinase domains. The binding to the box1 of GHR occurs through the FERM domain (93). Normally JAK2 is held in an inactive conformation, where the kinase and pseudokinase domain interact with each other (94). The activation of JAK2 requires that two catalytic domains are brought in close proximity. This was concluded after realizing that often in human leukemia there is oligomerization of JAK2 molecules which renders them constitutively active; this aggregation is due to the occurrence of a genetic fusion between the JAK2 catalytic domain and the oncogenic transcription factor TEL (95). Analysis of other mutation also contributed to the understanding of JAK2 physiology. The mutation V617A, which turns JAK2 into a constitutively active state, is found in patients with myeloproliferative disorders (96). This mutation probably disrupts the inhibitory interaction between the pseudokinase and kinase domain (97). Following mutational analysis, the SH2-pseudokinase domain linker turned out to be important for JAK2 activation (98). JAK2 activation results in the phosphorylation of multiple tyrosines. Several of these have been identified as important in the regulation of JAK2 function. For example, phosphorylation at tyrosine 1,007 is thought to expose the substrate and/or ATP binding sites (99), and phosphorylation of tyrosine 119 is thought to promote JAK2 dissociation from its receptor (100). Phosphorylation of tyrosine 813 appears to enhance JAK2 activity (101), whereas phosphorylation of tyrosine 221 decreases it (102). The importance of many of the JAK2 tyrosines is related to their roles in recruiting ancillary molecules needed for signaling propagation or signal termination.

Unexpectedly, the JAK/STAT signaling pathway is downregulated at febrile temperatures (103). JAK2 protein levels rapidly decrease in cells exposed to thermal stress, while its synthesis remains normal. The analogy of these findings in a variety of cell lines, as well as in PBMCs isolated from human blood, indicate the universal validity of this effect. Although JAK2 is a stable protein, it is degraded in a ubiquitin-dependent manner *via* the ubiquitin proteasome pathway (104). The significance of this process was illustrated in mouse 3T3 cells that showed a decreased GH response at 40°C. JAK2 underwent aggregation in an irreversible manner. Interestingly, kinase-inactive JAK2 did not show aggregation, although the effect of degradation in the cytoplasm at elevated temperatures was conserved. The findings predict that elevated body temperatures lower the responsiveness of cytokine receptors.

### SRC Activation

For a long time JAK2 has been regarded as the only kinase activated directly *via* the GHR. However, recent data, indicating that not all the GH signaling events rely on JAK2, brought controversy to the field. In particular, the activation of Lyn (SRC family kinase member) can occur independently of JAK2. First evidence came from a study by Zhu et al, who showed this by using pharmacological inhibitors and kinase inactive proteins (105). Additional evidence came from Rowlinson and co-workers, who reported that interfering with the GH-induced GHR conformational change affects JAK2 and Lyn activation differently (85). Activation of STAT5b by GH seems to require only JAK2, while activation of the small GTPases RalA, RalB, Rap1 and Rap2 by GH requires both Lyn and JAK2 (105). Lyn activation by GH was shown to activate MAPKs, also referred to as extracellular signal-regulated kinase 1 and 2 (ERK1 and ERK2), through the phospholipase Cγ-Ras pathway, signaling that might promote oncogenesis (85). Genes exclusively regulated by Lyn include genes involved in mRNA transcription and metabolism, including the GHR itself: the basal GHR expression level *via* Lyn is 4.8-fold higher, comparing GHR Box1-/- vs. GHR-/- (106). GHR signaling *via* this pathway induces also HLA-G, a powerful immunosuppressive protein for NK cells and macrophages. GH-enhanced immunosuppression in tumors might evade immune attack. On the other hand, it might be used to stop excessive inflammation after partial hepatectomy allowing liver regeneration and survival, Figs. 4 and 7 (107). For 3T3-F442A preadipocytes and H4IIE hepatoma cells it has been shown that relative levels of JAK2 and SRC family kinase in any particular cell might determine which kinase is the major signaling element, with JAK2 predominating in most cases (108). Barclay and co-workers showed that targeted mutation in the box1 of GHR in mice, although abrogating JAK2 activation, did not decrease the hepatic activation of MAPK *via* Lyn (106). The importance of this pathway came from studies with exon 3-deleted GHR, which results in the deletion of 22 amino acids in the extracellular domain of the GHR. Males with this genotype exhibit reduced basal but enhanced ERK signaling after GH stimulation. Exon 3-deleted GHR individuals showed lower serum IGF-1 levels, and were found to be of higher stature with extended lifespan (10 years) (109).

### Signaling Pathways of GH

The main pathways activated by GH are: the signal transducer and activator of transcription (STAT) pathway, the mitogen-activated protein kinase (MAPK) pathway, and the phosphoinositide-3 kinase (PI3K) pathway (**Figure 4**). The extent by which each pathway is activated depends on the cell types, related to differences in relative expression levels of the components of each pathway.

#### The STAT Pathway

STATs are latent transcription factors that upon activation by certain hormones or cytokines undergo tyrosine phosphorylation in the cytoplasm, dimerize *via* phosphotyrosine-SH2 interactions, and translocate into the nucleus where they activate transcription of specific genes (110). In mammals seven members of STAT have been identified with molecular weights ranging from 95 to 111,000 Mr (111). GH stimulation creates STATs binding sites in the GHR-JAK2 complex. The activation of STAT5b is critical for many of the GH biological functions, including metabolic changes, body growth and sex-dependent liver gene regulation (112, 113). Sex-biased genetic programs in liver metabolism and liver fibrosis are controlled by EZH1 and EZH2 downstream of GH-activated STAT5b (114). STATs 1 and 3 also become activated in response to GH (112), but their importance is still unclear.

STAT5b binds to the promoter elements of the IGF-1 gene, regulating its transcription in a GH-dependent manner (115, 116). A mutation in STAT5b, affecting its GH-induced tyrosine phosphorylation, caused severe growth retardation and immunodeficiency in one patient (117). Since then, more germline STAT5b missense variants with demonstrable dominant-negative effects, associated with short stature and mild immune dysregulation were identified in three unrelated families (118). This reiterates the importance of STAT5b for IGF-1 expression. STAT5b, but not STAT3, requires an intact and tyrosine phosphorylated GHR cytoplasmic tail for full activation (119). The key GHR tyrosines necessary for this event were identified (120, 121).

#### The MAPK Pathway

The Ras/MAPK, or ERK/MAPK has also been shown to be activated by GH. GHR phosphorylation creates docking sites for Src homology 2 domain-containing transforming protein C (Shc) (122). Shc gets then phosphorylated by JAK2, and binds growth factor receptor-bound protein 2 (Grb2) which binds Son of Sevenless (SOS), a guanine nucleotide exchange protein. Subsequently, Ras, Raf, mitogen-activated protein kinase/extracellular-regulated protein kinase (MEK), and ultimately MAPKs are sequentially activated (123). Phosphorylated MAPKs translocate to the nucleus where they transactivate transcription factors, and change gene expression to promote growth differentiation or mitosis. Data suggest that GH-dependent activation of the Ras/MEK/MAPK pathway contribute to GH-stimulated c-fos expression through serum response element (SRE). It remains controversial whether and how MAPK activation affects GH-induced proliferation and anti-apoptosis (124). As explained above, the activation of MAPKs may occur in a Lyn-dependent, JAK2 independent way. As STATs are also serine phosphorylated for full activity (125), it was suggested that this is mediated by MAPK pathway (126).

Some evidence suggests that GH signaling *via* MAPK pathway may engage in cross-talk with signaling pathways induced by other stimuli. Yamauchi and co-workers propose an interesting mechanism by which GH activates MAPK through stimulating the phosphorylation of a Grb2 binding site in the epidermal growth factor (EGF) receptor (127). Additionally, studies by Kim and co-workers show that GH stimulation alters the phosphorylation status of ErbB-2, a tyrosine kinase growth factor receptor member of the EGF receptor family, in a MAPK dependent manner (128). GH has also been described to activate other members of MAPK pathway, namely p38 MAP kinase and c-Jun amino-terminal kinase (JNK) (129, 130).

#### The PI3K Pathway

GH has also been shown to stimulate the PI-3K pathway, probably through tyrosyl phosphorylation of the large adaptor proteins, the insulin receptor substrates (IRS). GH stimulates the phosphorylation of IRS-1, -2, and -3 by JAK2, which leads to their association with multiple signaling molecules including the p85 subunit of PI-3 kinase (122, 131). This pathway is shared by the insulin signaling pathway, which may justify the insulin-like effects of acute GH stimulation under certain conditions, as discussed above. Particularly, activation of PI-3 kinase mediates the GH-induced increase in glucose transport, *via* induction of translocation of the glucose transporter 4 (GLUT4) to the cell surface (132), and has been suggested to mediate the ability of GH to stimulate lipid synthesis (133, 134). Additionally, PI-3 kinase activation results in AKT activation, which has been implicated in GH-promotion of cell survival. Activation of AKT depends on JAK2 binding to box1 in the GHR (135). Another kinase, p70S6K, involved in the control of cell proliferation and differentiation was shown to be activated by GH through PI-3 kinase and protein kinase C (PKC) (136). The NFκB pathway has also been shown to be activated by PI3-K and downstream AKT after GH stimulation (137).

### GH Desensitization

Termination of the GHR signaling is an important mechanism for controlling GH actions (**Figure 4**). Protein tyrosine phosphatases (PTPs) are employed by the cells for negative regulation of GH signaling, namely SH2 domain-containing protein-tyrosine phosphatase (SHP-1), SHP-2, protein-tyrosine phosphatase (PTP)-H1, PTP1, TC-PTP, and PT1b (138). Mice, deficient in SHP-1, have prolonged JAK-2 phosphorylation and STAT5b activity, which represents strong evidence for an important role of this phosphatase in the deactivation of GH signaling (139). There are conflicting reports concerning the physiologic importance of SHP-2 in GHR: while Frank et al. concluded that SHP-2 is a positive regulator (140), Stofega et al. proposed SHP-2 as an inhibitor of GH signaling (141). GH stimulation has been shown to trigger the phosphorylation of JAK2-associated SIRP-α, signal regulatory protein alpha. This was suggested to promote SHP-2 recruitment and consequent attenuation of GH signaling (142). A study by Pasquali has identified PTP-H1, PTP1, PTP1b, and TC-PTP as specific interactors of phosphorylated GHR (143). PTP1b knockout mice display increased JAK2 and STAT5b phosphorylation, while PTP-H1 knockout mice display enhanced growth (144, 145). CD45 was shown to be a JAK2 phosphatase, being able to suppress its activity and regulate cytokine receptor signaling (146).

Other regulators are PIAS, “protein inhibitors of the activated STATs”, which display SUMO ligase activity. PIAS can bind STAT proteins, and prevent their association to the DNA. Although the majority of the PIAS interactor proteins are prone to modification by SUMO, the exact mechanism by which PIAS influences STAT5b function is still unclear (147). Some studies have also implicated the adaptor protein Grb10 as regulator of GH signaling. Grb10 interacts with GHR upon GH stimulation, and downregulates GH signaling pathways downstream of JAK2 and independently of STAT5b (148). Work of Carter-Su and colleagues found that SH2B-β association with JAK2 enhances its activity (149). Thus, decrease in SH2B-β levels could contribute for GH-signaling termination. Other cellular factors that modulate GH sensitivity are insulin, thyroid and sex hormones, as well as inflammatory cytokines (150, 151).

In addition to direct interference with the signaling molecules, cells have the capacity to tune the number of GHRs at the cell surface in several ways. As described above, the extracellular domain of GHR can be cleaved in a process called shedding. One of the consequences of this process is the reduction of the number of signaling competent receptors at the cell surface, and consequent regulation of the cell sensitivity to GH (124). Since GH binding to GHR inhibits its shedding, this mechanism cannot be regarded as signal terminator (152, 153). However, the most powerful and best studied mechanism to control GH sensitivity of cells is endocytosis. Opposite to other type 1 cytokine receptors, GHR is endocytosed both in the presence and absence of ligand (154, 155). Therefore, besides regulating the responsiveness of the cells to GH, endocytosis of GHR provides a very efficient way for GH signaling attenuation. The next paragraph will be dedicated to the advances made in understanding GHR endocytosis.

## The Ubiquitin System in Receptor Trafficking

Ubiquitin is a small molecule of 76 amino acids which C-terminus is attached to proteins upon sequential action of three enzymes: a ubiquitin activating enzyme (E1), a ubiquitin conjugating enzyme (E2), and a ubiquitin protein ligase (E3). Ubiquitin may be added as a single monomer or multiple monomers, or as a polyubiquitin chain. The addition of ubiquitin to target proteins covers a great variety of functions. Endocytosis is the main way used by the cells to achieve the homeostatic regulation of plasma membrane protein abundance. Once a protein is endocytosed it is either recycled back to the cell surface or captured in the intraluminal vesicles of the MVBs, and eventually guided to lysosomes for degradation (156).

Ubiquitination has emerged as a central mechanism governing the subcellular trafficking of proteins, reviewed in (7). It is critically important for the regulation of the number of receptors and transporters at the plasma membrane. The first evidence for a role of ubiquitin in the membrane trafficking came from the work of Kölling and collaborators with the ABC-transporter Step6 in yeast (157). In mammalian cells, the first receptor reported to depend on the ubiquitination system for its endocytosis and degradation was GHR (3). From then on, many more receptors were shown to depend on the ubiquitin system to be endocytosed, often in response to ligand binding (7, 158).

Ubiquitination works as an engagement tool of the proteins with the endosomal sorting complexes required for transport (ESCRTs) (159). In fact, ubiquitination has been reported at several points along the endocytic pathway. Although monoubiquitination has been regarded as a sufficient signal for sorting, K63 linked polyubiquitin chain are now considered as the primary sorting factor. Studies with the GAP-1 permease indicated that monoubiquitination is sufficient for initial internalization, but further sorting in the endosomes requires K63-linked polyubiquitin (160). Also studies of the mammalian TrkA and MHC class 1 proteins showed the importance of this type of chains in their MVB sorting (161, 162). Within the endocytic system, ubiquitin acts as an interaction module that is recognized by a variety of ubiquitin binding domains (UBDs), including UIM, CUE, NZF, and certain VHS and SH3 domains present in several proteins (163). As illustrated in **Figure 2**, after endocytosis, the next step in the sorting route is the selection by the ESCRT-0 complex, which acts at a branch point in endosomal traffic: binding to certain cargo (like the GHR) commits it to degradation in the lysosome, while cargo that does not bind (like transferrin and Low Density Lipoprotein receptors will be recycled to the plasma membrane. ESCRT-0 is composed of HRS and STAM, both of which bearing UIM (ubiquitin interacting motif) and VHS (Vps27, HRS and STAM) domains, important for ubiquitin binding and cargo recognition (164–166).

**Figure 5:**
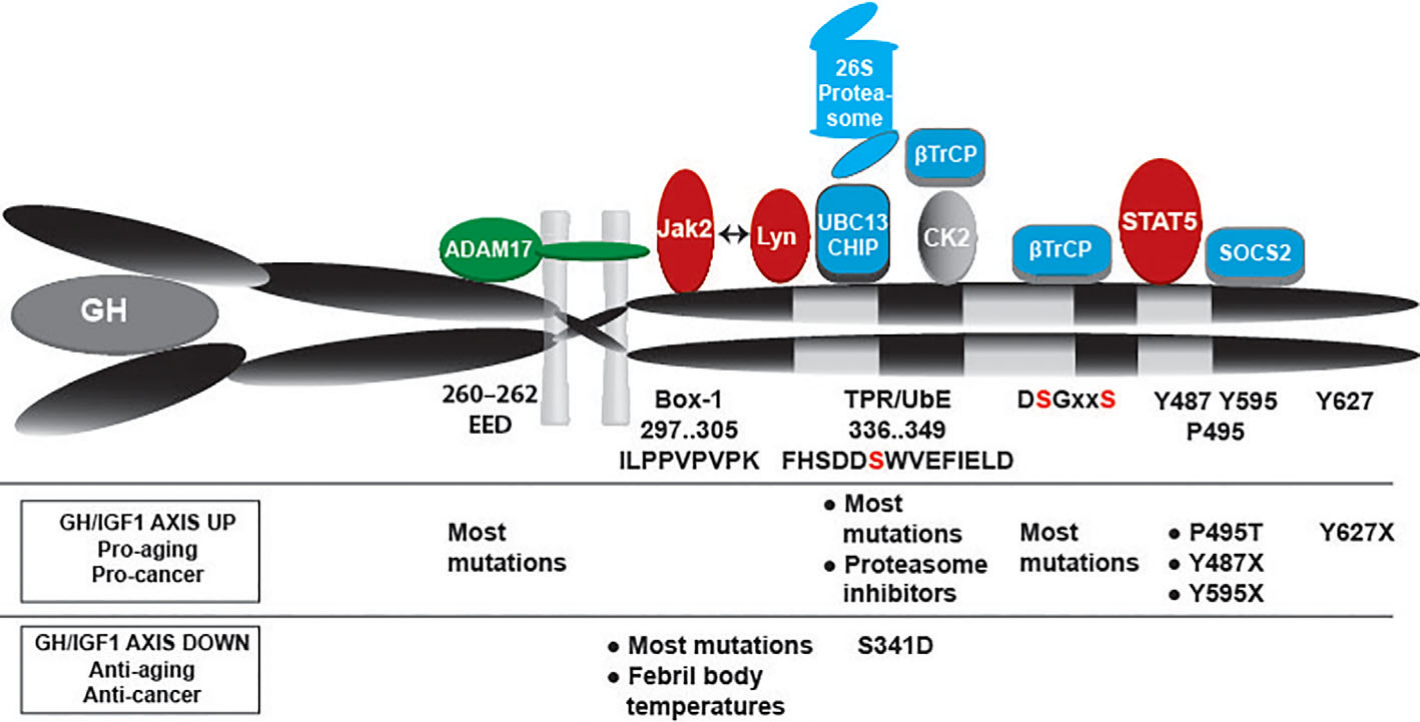
GHR controlling factors: ADAM17, JAK2, Lyn, Ubc13/CHIP, proteasome, βTrCP, STAT5b, SOCS2 and possibly CK2. Their respective bindings sites are indicated. JAK2 and Lyn bind to the same membrane-proximal region, with overlapping substrate binding sites. Under basal conditions βTrCP binds to the constitutively phosphorylated DSGxxS sequence. The kinases responsible for DSGxxS phosphorylation are unknown. Under conditions of GH stimulation JAK2 is released from the receptor, and the serine 341 of the UbE motif gets phosphorylated probable by CK2. These events increase the affinity of βTrCP for the UbE motif, reduce the role of the DSGRTS sequence, and recruit STAT5b and SOCS2 to their respective binding sites (pY containing motifs). Overall, GH stimulation shortens the residence time at the plasma membrane and results in faster GHR endocytosis. The lower part indicates the effects of mutations and other conditions that might impact the GH sensitivity of cells: increased GH/IGF-1 activity acts pro-aging and pro-cancer, while lower activity acts anti-aging and anti-cancer.

Other important components to consider in the endocytic regulation are the deubiquitinating enzymes (DUBs), which are specific proteinases able to remove ubiquitin moieties from proteins. Besides the catalytic domain, DUBs contain domains that allow them to associate with scaffolding proteins and adaptors. The ESCRT machinery associates with at least two DUBs: AMSH and USP8 (UBPY). In yeast, Doa4 has been identified as an additional DUB, important for receptor recycling. Deubiquitination of endocytosed receptors before or after delivery into the MVBs may profoundly affect receptor trafficking, and ultimately substrate turnover rate (167). It remains unclear how the ubiquitinated cargo is handed from one sorting step to the other. Models have been put forward based on a gradient of sorting proteins containing ubiquitin binding domains of increasing binding affinities. More complexity can be added to this model if we consider ubiquitin ligases such as Triad1 and DUBs along the sorting pathway, which could perform additional chain editing (156, 168). In **Figure 5**, the different controlling factors are depicted with reference to their effects on residence time at the cell surface and consequences of loss of function for the GH/IGF-1 axis.

### Role of SCF^TrCP1^ in GHR Endocytosis

The SCF (SKP1-CUL1-F box protein) subfamily of E3 ligases was originally discovered and studied in budding yeast *Saccharomyces cerevisae* (Patton et al., 1998). They are the best characterized mammalian cullin RING ubiquitin ligases. The determination of the crystal structure of SCF complex by Zheng and co-workers added some insights in the roles of each of its components and the mechanistic aspects of their interlinked actions (Zheng et al., 2002; Frescas and Pagano, 2008).

The endocytosis and degradation of GHR depends on the ubiquitin conjugation system, as shown for the first time in a Chinese hamster lung cell line (ts20) with a temperature-sensitive mutation in the E1 enzyme. Whereas at the permissive temperature the endocytosis of GHR occurred normally, when the cells were put at non-permissive temperature the GHRs accumulate at the cell surface (3). Further evaluation revealed that GHR ubiquitination and clathrin dependent-GHR endocytosis are coupled events (40, 41). An important achievement in the mechanistic understanding of GHR endocytosis was the discovery of the “Ubiquitin-dependent endocytosis motif”, UbE, 340-349, which consists in the amino acid sequence DSWVEFIELD (169). If this motif is mutated the ubiquitination and endocytosis of GHR are strongly inhibited. Besides this motif, there is a di-leucine motif at the position 365-366. This motif mediates fast ubiquitin-independent, clathrin-dependent GHR endocytosis only if the receptor is truncated at position 367, probably due to its complete exposure in this case. The functionality of the di-leucine motif in the context of full-length receptor is not apparent (170). Surprisingly, a GHR truncation (at amino acid 417), where all its lysines were mutated to arginines, although not being ubiquitinated, was normally endocytosed in a ubiquitin-system dependent manner. This indicates that the ubiquitination of GHR itself is neither needed for its endocytosis nor for its degradation (169). One reason that justifies the importance of the UbE motif in GHR endocytosis is its binding site for the SCF^βTrCP^ E3 ligase (171). The role of the UbE motif and βTrCP has been also extended to sorting at the MVB and degradation at the lysosomes (172). JAK2 was also identified as a stoichiometric regulator of GHR endocytosis. Besides its role in signaling, merely binding of JAK2 to GHR is inhibitory for its endocytosis. As many cytokine receptors are JAK2 clients, cellular levels of JAK2 play a role in cytokine sensitivity, best illustrated by its sensitivity to febrile temperatures (103). The model is that GHR can only be endocytosed if JAK2 has detached from it, which was proposed to occur after GH stimulation. It is possible that JAK2 binding/dissociation cycles have direct effects in the ubiquitination events mediated by SCF^βTrCP^, and thereby affect rate of GHR endocytosis (92). Not surprisingly, the life cycle of JAK2 is controlled by E3 ubiquitin ligases of the CBL family as has been shown in hematopoietic stem cells and myeloid malignancies (173).

The UbE motif works as a recruitment platform for βTrCP, the F-box substrate recognition subunit of SCF^βTrCP^ E3 ligase, necessary for GHR endocytosis (171). Generally, SCF^βTrCP^ recognizes the classical DSGxxS motif in its substrates (174), including receptors homologous to GHR, such as prolactin receptor (PRLR) (175), interferon-α receptor (IFNAR) (176), and erythropoietin receptor (EpoR) (177). In these receptors, βTrCP binds only upon ligand binding when the serine residues in the DSGxxS motif are phosphorylated, which leads to their endocytosis and signal termination. The GHR also contains a D^383^SGxxS motif. This is constitutively phosphorylated, able to bind βTrCP, and contributes to the steady state endocytosis of the GHR (the half-life of unligated (mature) GHR is 30 min) (39, 42). Therefore, in contrast with other cytokine receptor family members, GHR DSGxxS motif does not seem to contribute to signal termination. This role is carried out by the UbE motif, important for both steady state and GH-induced endocytosis (42, 92). NMR experiments demonstrated that the UbE motif is essentially unstructured, and, together with functional mapping of the UbE and βTrCP revealed a unique interaction model of βTrCP with GHR-UbE (178). Since the regulation of βTrCP-substrates interactions involves serine phosphorylation, we evaluated the potential role of the UbE serine phosphorylation (S341) as a modulator of UbE-βTrCP interaction. Binding studies comparing affinities of the interaction of βTRCP to unphosphorylated vs phosphorylated S341 peptides (Surface plasmon resonance and pulldowns) showed 100 times increase in binding affinity upon S341 phosphorylation. Accordingly, ^125^I-GH binding/internalisation assays in cell lines stably expressing S341A or S341D (phosphomimetic) suggest that GH stimulation triggers faster GHR endocytosis by causing phosphorylation of S341 in the GHR UbE motif and subsequent increase in UbE-βTrcP binding affinity (179). S341 phosphorylation might constitute a very efficient mechanism for signal termination after GH stimulation.

The kinase responsible for S341 phosphorylation is unknown yet. S341 is contained in a minimal consensus site for CK2 phosphorylation, which has been identified to be S-X-X-Acidic. The acidic residue may be glutamate, aspartate, or phosphorylated serine and tyrosine: in case of S341 this sequence is S^341^WVE (180, 181). Preliminary studies on the evaluation of a potential role for CK2 in S341 phosphorylation, by using the CK2 inhibitor 4,5,6,7-tetra-bromo-benxotriazole (TBB) (182), revealed that CK2 is a promising target. It has become apparent that the regulation of CK2 involves regulated expression, assembly and subcellular localization, post-translational modifications, and regulatory interactions with molecules and proteins (183, 184). Interestingly, there are reports of increased CK2 activation by insulin, EGF, IGF-1 (185, 186) and TNF (187, 188). EGF-activated ERK2 binds directly CK2α enhancing its activity (189). TNFα-induced activation of CK2 was also related to ERK1/2 activity (187). It is interesting to evaluate whether CK2 can be activated by GH. Other stressors or pathways that activate ERK1/2 may result in increased activity of CK2 towards S341 in GHR, resulting in increased GHR endocytosis. Future studies will elucidate this hypothesis.

### The SOCS Family and GHR

The suppressor of cytokine signaling (SOCS) family of proteins plays a very important role in the GH-signal termination. This family is composed of eight members, and the expression of four of them is stimulated by GH, namely SOCS1, -2, -3, and CIS (cytokine inducible SH2-constaining protein) (138, 190). Structurally, SOCS proteins contain a central SH2 domain and a motif called SOCS box at their C-termini (138). The SOCS box mediates the formation of multi-subunit ubiquitin ligases, containing elongin BC, cullin 2 or 5 and the RING finger proteins Rbx1 or Rbx2 (191). SOCS1 and SOCS3 contain an additional kinase inhibitory region at their N-termini (KIR). Different SOCS apply different mechanisms for GH signaling downregulation. SOCS1 is thought to bind Y1007 on JAK2 activation loop, and by doing so, to inhibit JAK2 activity through KIR (192). SOCS3, besides binding to the same residue in JAK2 (193), also binds to phosphorylated tyrosines in GHR. Also, SOCS2 and CIS have been shown to bind to phosphorylated GHR, which was suggested to interfere with STAT5b-GHR binding (194). SOCS1, and possibly SOCS2 and SOCS3 use their ubiquitination activity to mediate GHR and JAK2 degradation and, therefore, signal termination (21, 104, 138). A role of CIS as a stimulator of GHR internalization and proteasomal degradation has been proposed (195). Interestingly, there is evidence that some stimuli that reduce GH sensitivity, such as estrogen or sepsis, do so by increasing expression of certain SOCS proteins (196, 197). The physiological importance of SOCS in GH signaling regulation is unclear since SOCS1^-/-^, CIS^-/-^ and liver specific SOCS3^-/-^ are not bigger than normal (198–200). Only SOCS2 knockout mice are larger than wild-type (201). SOCS2 inhibits the GHR *via* binding to pY487 and pY595 (202, 203). SOCS may play a key role in shifting GH action from growth-promotion to lipolysis. Two independent studies showed that a single-nucleotide polymorphism in GHR resulting in a P495T substitution was associated with lung cancer (204, 205). Y595 (and Y487) were previously indicated as a binding site for the phosphatase SHP2 (141) Recently, Chhabra and collaborators showed a causative relation with SOCS2 binding to the GHR in which both P495 and pY487 are required (21). They show that GH-induced signaling increased AKT pT308 signaling significantly in GHRP495T cells. This is a strong prognostic indicator for non-small cell lung cancer (206). In addition, STAT3 was activated. Activated STAT3 is an important oncogenic factor during carcinogenesis and metastasis of both small cell lung cancer and squamous cell lung carcinoma (207). Taken together, SOCS family members, especially SOCS2, play an important role in the regulation of the GHR.

### CHIP and GHR

In an effort to identify additional ubiquitination factors involved in the fate of GH receptors we used a small siRNA library targeting a selection of ubiquitination factors (35). As K63-linked ubiquitin chains have been implied in the regulation of membrane receptor trafficking, we search for such factors (160, 208). Silencing of the ubiquitin conjugase (E2) UBC13 came up as a GHR-specific endocytosis factor (165, 209). Previously, pull-down experiments showed that the UbE motif has an affinity for tetratricopeptide repeat-containing (TPR) proteins (30). As UBC13 can serve as E2 for a ubiquitin ligase that binds substrates *via* its TPR motif we tested both (C-terminus of Hsp70 interacting protein) CHIP and UBC13 for endocytosis and degradation of the GHR: both factors were required and collaborated in GH-induced endocytosis of the GHR (209). Using blue native electrophoresis, Sedek et al. confirmed K63-linked proteins in large GHR-containing protein complexes stimulated and isolated through streptavidin pull-down during endocytosis (27).

CHIP is an E3 ubiquitin ligase that plays a pivotal role in the protein quality control system by shifting the balance of the folding-refolding machinery toward the degradative pathway in order to maintain balanced proteostasis networks (33, 210–212). CHIP is highly expressed in tissues with high metabolic activity and protein turnover. In addition, as a regulator of growth and metabolism, CHIP mediates monoubiquitination and subsequent endocytic-lysosomal turnover of the insulin receptor (INSR). CHIP deficiency results in increased INSR levels that lead to premature aging in various organisms. The detrimental effects of the increased INSR level are mainly due to a PI3K/AKT signaling (213). In line with this, CHIP ubiquitinates AKT independent of its phosphorylation state (214). Remarkably, transcription of CHIP is also modulated in response to changes in AKT levels (215). Similar to AKT regulation, CHIP indirectly impacts the FOXO function on various levels through modulation of upstream substrates of the insulin/IGF-1 signaling pathway, a pivotal genetic program regulating cell growth, tissue development, metabolic physiology, and longevity of multicellular organisms (210). Thus, CHIP integrates proteostasis and aging by regulation the turnover of the INSR (211). In cancer the pathogenic mechanisms of CHIP are less clear (216). Several studies on breast cancer cells have indicated CHIP as tumor suppressor (212, 217–219).

## Physiological Roles of GH

### Promotion of Growth

The promotion of postnatal growth is a major physiological function of GH. Initially, it was thought that GH indirectly stimulates growth *via* triggering the production of IGFs, or somatomedins, exclusively in the liver. This was called the “somatomedin hypothesis”. This theory was challenged when direct actions of GH on several peripheral tissues were reported (220). In fact, liver-specific IGF-1 gene-deleted mice show normal postnatal growth and development despite the low levels of IGF-1 in circulation. This indicates that direct effects of GH in target tissues (adipose tissue, bone and skeletal muscle) are involved in growth promotion, and probably in stimulation of local IGF-1 production (221). According to the dual effector hypothesis of Green et al. GH may stimulate early recruitment of stem cells followed by further clonal expansion due to GH-induced IGF1 expression (222). The critical importance of GH as the main endocrine mediator of growth is proven either by the dwarf phenotype occurring when the levels of GH are insufficient during early development, or by gigantism, due to hyper-secretion of GH before puberty (53). Apart from GH secretion, also defects in GHR and post-receptor signaling may result in phenotypes similar to GH hypo-secretion. Laron and coworkers described for the first time the clinical phenotype of severe growth defect, and for that reason it is named “Laron Syndrome” (223). Deletions and mutations in GHR have been described as causative for this phenotype (224, 225). Studies on a large cohort of individuals in northeastern Brazil who were homozygous for a mutation in the GH-releasing hormone receptor gene revealed comparable phenotypes. These individuals are characterized by severe dwarfism, due to very low GH and IGF-1 levels, increased adiposity, and increased insulin sensitivity (226). Lean body mass (LBM) is reduced, but muscle function is adequate. Their longevity and quality of life are normal, and they are largely protected from cancer and less prone to atherosclerosis (227).

Postnatal growth of mice has been shown to rely on signaling mediated by JAK2 and STAT5. GHR-/- mice with knock-in GHR1-391, which eliminates all GH-mediated STAT5b signaling while still allowing activated hepatic JAK2 and ERK2, showed substantially decreased growth (228). This study identified many genes as STAT5b-regulated, such as IGF-1, Igfals, Socs2, P450 cytochrome, Cyp2b9, and some metabolic enzymes. Eleven of these were upregulated (e.g., Sth2, Hao3, and Ang), and nine were downregulated (e.g., Igfals, IGF-1, EgfR, and Comt). These results confirm the importance of STAT5b in growth promotion. On the other hand, pituitary adenomas that cause hypersecretion of GH result into excessive growth called gigantism when present before puberty, whereas in adulthood it results in a clinical condition called acromegaly (229). In these patients excess of GH, besides affecting the size of hands, feet, and fingers, has important metabolic consequences, suggesting additional functions for GH, that will be discussed next in this review.

### Metabolic Regulation

**Figure 6:**
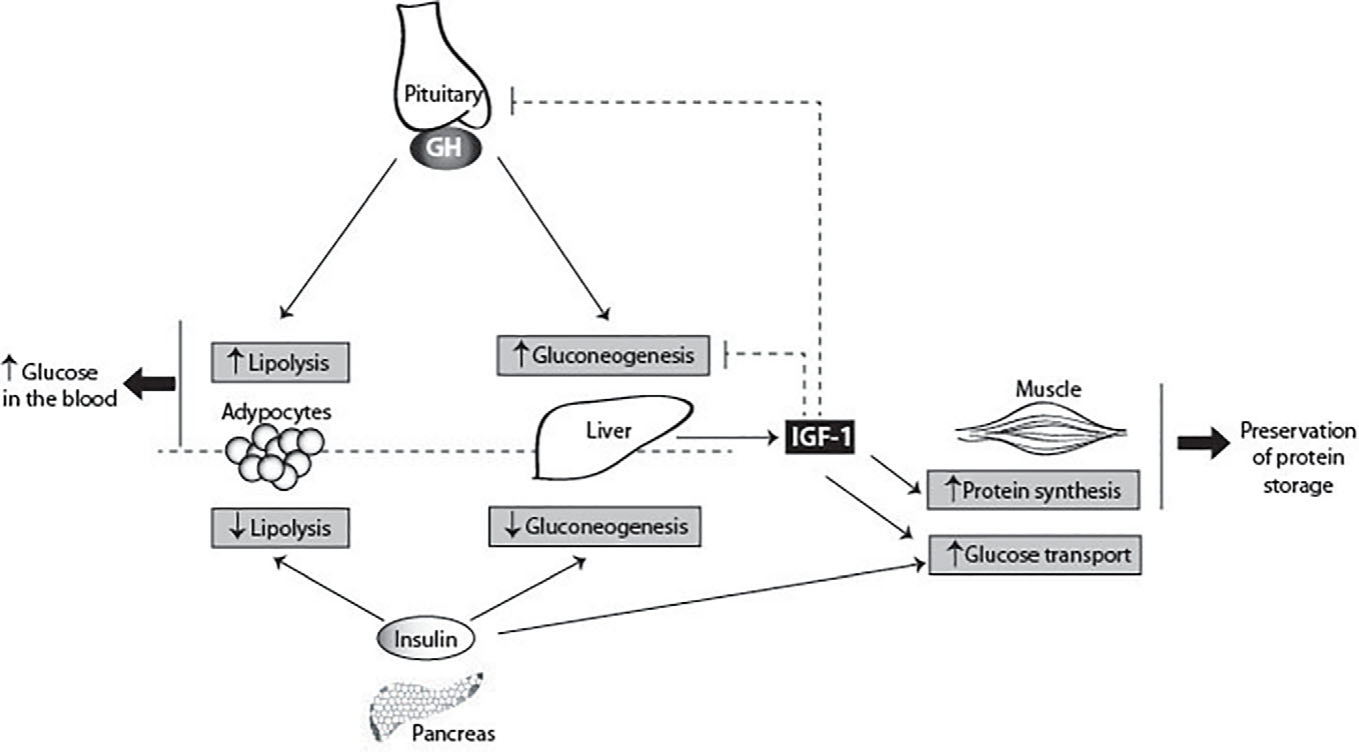
GH metabolic actions. GH has pleiotropic effects on carbohydrate, lipid, and protein metabolism. GH antagonizes the effects of insulin, secreted by the pancreas, by two direct ways: inhibiting gluconeogenesis in the liver, and increasing lipolysis in adipocytes. GH also stimulates the production of IGF-1 by the liver. IGF-1 suppresses gluconeogenesis in the liver *via* the insulin receptor. In the muscle, IGF-1 stimulates directly glucose uptake, and stimulates protein synthesis. IGF-1 inhibits GH secretion by the pituitary gland, and therefore indirectly blocks the insulin antagonizing effects of GH, contributing for the glucose homeostasis. The main consequences of GH metabolic actions are the increase of glucose levels in the blood and preservation of protein storages.

GH holds important roles in metabolic regulation (**Figure 6**). As soon as human pituitary extracts became available it was shown that injection of large amounts of GH both in healthy subjects and GH-deficient patients stimulated lipolysis and led to hyperglycemia (230–232). Indeed, as expected, hyper-insulinemia, impaired glucose tolerance, and overt diabetes mellitus are common features of acromegaly (233). GH works as a metabolic switch between carbohydrate and lipid utilization: in conditions of energy surplus GH acts in concert with IGF-1 to promote nitrogen retention, while during starvation GH switches fuel consumption from carbohydrates and protein to lipids. This guarantees the preservation of protein stores and consequently maintains LBM. The direct acute metabolic effects of GH in the basal state are the stimulation of lipolysis and the consequent increase of free fatty acids (FFA) in the blood. Repetitive GH pulses in presence of adequate energy supply and concomitant increased insulin levels induces IGF-1 production (234, 235). Consequently, in the long range, protein stores and LBM increases, while body fat mass decreases. GH stimulates the cell growth of the skeletal muscle by facilitating myoblasts fusion. Like in more peripheral tissues GH does not regulate IGF-1 expression in myotubes. On the other hand IGF-1 has been implicated in skeletal muscle hypertrophy, attenuation of age-related skeletal muscle atrophy, and restoring and improvement of muscle mass (236, 237).

Studies evaluating the acute effects of GH on protein metabolism in the basal state have produced inconsistent conclusions. While some studies indicated that acute GH stimulation leads to increased muscle protein synthesis, others did not detect any effects of GH withdrawal in protein metabolism (238). Less controversial are the studies evaluating the effects of GH on protein metabolism in pathological states (acromegaly and GH deficiency) and in stress (exercise and fasting). Stress conditions are the natural domains of GH, in which the body benefits from GH effects on substrate metabolism (238). During fasting GH is the only anabolic hormone to increase (239). GH administration has been shown to be beneficial for protein preservation in conditions of dietary restriction (240). Moreover, fasting in GH-deficient subjects resulted in 50% increase in urea-nitrogen excretion and 25% increase in muscle protein breakdown (241, 242). Also, obesity has been associated with decreased levels of circulating GH, and consequent protein loss (243). Treatment of these patients with GH has been successful in preserving the protein stores and LBM (244). Although the metabolic functions of GH are well recognized, the underlying mechanisms of these actions are not yet well described.

Under certain conditions (e.g. if cells are deprived of GH for some hours), GH has acute and transient insulin-like effects (134). These effects include increased glucose utilization, increased glucose uptake, anti-lipolysis among others. It has been suggested that these effects are mediated by the insulin receptor substrate-1 (IRS-1) and phosphoinositide 3-kinase (PI3-K), which get activated by GH stimulation (245).

There is an extensive interest in taking advantage of the anabolic effects of GH for improving athletic performance (246). During moderate exercise GH appears to stimulate lipolysis without any effect on protein and glucose metabolism. Prolonged GH administration results in prevalent lipolysis and decreased protein oxidation (238). Although administration of supra-physiological doses of GH to athletes exerts potentially beneficial effects on body composition, it remains unclear whether these effects translate to improved performance (247). Nevertheless, GH abuse has been widespread among the athletes for more than 20 years, with consequences such as edema, carpal tunnel syndrome, arthralgias, myalgias, glucose intolerance and diabetes mellitus (248, 249).

### Roles in the Immune System

At the cellular level, GH stimulates differentiation and mitogenesis and prevents apoptosis (137, 145, 250). There are also reports that GH signaling results in tubulin polymerization (251), cell migration and chemotaxis (252). These cellular effects are implicated in a variety of biological actions of GH in immune cells. Both GH and its receptor are expressed in various immune cells as T lymphocytes, B lymphocytes, monocytes and neutrophils. GH enhances thymopoiesis and T cell development, modulates cytokine production, enhances B cell development and antibody production, activates neutrophils and monocytes, enhances neutrophil adhesion and monocyte migration, and it has an anti-apoptotic action (253). Additionally, GH is involved in the formation and functional activation of mature blood cells (254).

GHR clearly acts in favor of an active immune system. In aging people the immune response gradually deteriorates due to a downregulated GH/IGF-1 axis (238). This became particularly clear during the Covid-19 pandemic in 2020 when death rates were very high among elderly. Lowered GH/IGF-1 activity promote inflammatory activity, causing long term tissue damage and systemic chronic inflammation due to decreased levels of anti-inflammatory adipokines such as adiponectin (255). Somewhat counterintuitive, studies of long living mutants and caloric restricted animals show decreased pro-inflammatory activity with increased levels of anti-inflammatory adipokines such as adiponectin. This suggests that reduced inflammation promoting healthy metabolism may represent one of the major mechanisms of extended longevity in long-lived mutant mice and likely also in the human. Together it shows that there is a need for insight in the molecular mechanisms underlying the relation between the GH/IGF-1 axis and immunity. Recently, Ishikawa et al. found that induction of the mouse major histocompatibility complex (MHC) antigen blastocyst H2 (H2-Bl) expression by GH is critical for suppressing innate immune cells such as natural killer (NK) cells/NK T cells (NKTs) and macrophage-mediated hepatocyte apoptosis, which favors C57BL/6 mice liver regeneration and survival after partial hepatectomy. Application of human leukocyte antigen G (HLA-G, the human homolog of H2-Bl) gave similar results (107). They used a series of knock-in mice to prove that, rather than the JAK/STAT pathway, GHR signaling from SRC, presumably Lyn, bound to the GHR receptor, activates ERK *via* RAS (85, 106). Thus, H2-Bl expression is crucial for reducing innate immune-mediated apoptosis and promoting survival after partial hepatectomy. This is important progress on the long road to understand the relation between GH/IGF-1 signaling and immunity regulation.

### Roles in the Brain

The activity of GH/GHR in the brain is still a matter of debate. GH has been described as modulator of stress response and behavior by acting directly on the brain (256). Recent studies show that GH has direct trophic effects on the formation of proopiomelanocortin- and agouti-related peptide-expressing neurons and provide evidence that GH regulates hypothalamic neurocircuits controlling energy homeostasis (257). IGF-1 is well known to be critical for neuronal structure and function, and models of IGF-1 deficiency demonstrate important hippocampal deficits, as reduced structural complexity, excitability, learning, and memory (258–261). Interestingly, low IGF-1 levels have been associated with risk for vascular dementias (262). On the contrary, studies with growth hormone deficient (GHD) people and mouse models support the notion that a decrease in GH action is beneficial for maintenance of CNS integrity and functions as learning and memory during aging (263–265). Both in humans and rodents, circulating GH and IGF-1 levels decline with age, including in the central nervous system (CNS) (266). However, despite low overall GH/IGF-1 levels, old Ames dwarf mice have elevated levels of GH and IGF-1 in the hippocampus. Also, human subjects with Laron syndrome show improved rather than impaired memory (13). A likely explanation for the disconnect between IGF-1 and GH roles in the brain was provided by Sun et al. who demonstrated that hippocampal IGF-1 expression is not reduced in GH-deficient mice (267). Importantly, studies by Efstratiadis provided evidence that in contrast to hepatic IGF-1 expression, IGF-1 expression in the brain is not GH-dependent (268). GHD and GHR deficient mice, which have longer life expectancies than wild-type, also perform better on cognitive tasks (255). Furthermore, three prominent models of GH signaling disruption—Snell dwarf, Ames dwarf, and GHRKO mice—all suggest reduced GH is beneficial to the CNS. Ames dwarf mice, which carry a homozygous loss-of-function mutation at the Prop-1 locus, are deficient in GH, thyroid-stimulating hormone (TSH), and prolactin-producing cells in the adenohypophysis. Despite their smaller body size and unique phenotype, these mice have a significantly increased lifespan and maintain physiological and cognitive function at youthful levels longer than controls (269, 270).

### GH and Aging

Knockout mice for GHR (“Laron Dwarf”) and mice with mutations causing GH deficiency or resistance (“Ames dwarf”, and “Snell dwarf”, and “Little”) live longer than their genetically normal siblings (4, 227, 271–274). This extended longevity is remarkable and reproducible, ranging from 25% to over 60%. These long-lived mice present many signs and indicators of a healthy delayed aging process. These results would lead to the conclusion that GH normally released by the pituitary limits life expectancy, probably due to acceleration of the aging process. This conclusion is supported by reports showing that reduced levels of IGF-1, or mutations interfering with IGF-1 signaling also result in increased mice longevity (275, 276). As expected, transgenic mice with elevated levels of GH and IGF-1 live shortly and reflect characteristics of an accelerated aging process (277). Studies analyzing the influence of GH signaling and lifespan in several species have been performed. Exciting new findings come from numerous tissue-specific GHRKO mice and include the role of GH in pancreatic β-cells to stimulate insulin following glucose challenge, in weight loss regulation in ablated AgRP neurons and in glucose homeostasis in LepR neurons. In muscle GHR disruption enhances insulin sensitivity and extends lifespan, while adult-onset global disruption of GHR extends female lifespan, reviewed in (278).

While Besson and co-workers reported that individuals with congenital GH deficiency live shorter (279), others reported that GH-deficient/resistant subjects live long with decreased incidence of cancer, atherosclerosis and vascular pathology, in spite of being obese (11, 280, 281). These conflicting results may be connected to another key factor influencing the aging process: the insulin sensitivity. In mouse models, GH-deficiency is associated with insulin sensitivity (low levels of circulating insulin), while GH-deficient people are insulin resistant (high levels of circulating insulin). In these models, GH-deficiency allied with insulin sensitivity contributes to low blood glucose level. This biochemical feature has been negatively correlated with oxidative stress (282). Since oxidative stress is recognized as one of the major causes of aging (283), GH may influence the aging process by acting on oxidative stress pathways. Accordingly, Ames dwarf mice produce less metabolic oxidants, and have increased levels of anti-oxidants (284, 285). On the other hand, in GH-deficient humans, insulin resistance increases the oxidative damage (282), induces accumulation of visceral fat mass (286), and increases the risk of several age-related diseases (12, 210, 287). Thus, differences in the aging process between mice and humans suffering from GH-deficiency may be explained by their difference in insulin sensitivity.

Recent studies show physical and functional interactions of GHR with IGF-1R, which might strengthen its role in aging (and cancer). Although absent in hepatocytes, IGF-1R can serve as a component of the GH signaling pathway, modulating GHR’s signaling strength and allowing for more local heterogeneity of GH/IGF-1 actions (86). Not only the GH-induced IGF-1 expression in the liver, also its secretion is subject of regulation. Studies of the energy sensing liver kinase B1-AMP-activated protein kinase (LKB1–AMPKa1) pathway implicate this pathway in the IGF-1 secretion *via* the small GTPase, Rab8a: the LKB1–AMPK pathway inhibits IGF-1 secretion. How this relates to the tumor suppressor functions of LKB1 remains unclear. Independent of the LKB1−AMP−activity, the insulin sensitizer and anti−aging/cancer agent, metformin also inhibits IGF-1 secretion (288–290). Once again, these studies illustrate the complexity of the GH/IGF-1 axis for metabolism, in homeostasis, as well in aging and cancer.

In humans, during aging the GH/IGF-1 axis is down regulated (238). On one hand, this probably contributes to the effects of aging on body composition, skin characteristics and functional changes that decrease the quality of life. On the other hand, decrease in the amounts of GH with age may offer protection from cancer and other age-related diseases. Therefore, GH replacement is controversial as an anti-aging therapy and involves both benefits and risks (274, 291).

### GH and Cancer

There is an overwhelming number of studies that implicate GH/IGF-I in cancer growth. Organisms that lack GHR activity are virtually devoid of cancer (4, 292–299). In addition to the pituitary, GH is expressed in colon, prostate, lung, meningiomas and breast, where it binds the GHR to signal in a paracrine/autocrine fashion (21, 297, 300–303). Elegant experiments with rodents reveal an important role of GH in tumor development. Crossing GHR KO mice with mice predisposed to develop carcinomas significantly slowed down tumor progression (304). Additionally, GH deficient rats crossed with rats predisposed to prostatic cancer showed significantly reduced tumor incidence and burden (305). Interestingly, GH-deficient female rats are resistant to chemical induction of mammary carcinogenesis, whereas GH replacement restores the risk of tumor development (306). Intracellular (autocrine) GH promotes breast cancer cell transformation (292, 294, 301, 307, 308) and induces an invasive phenotype by triggering an epithelial–mesenchymal transition (EMT), cell motility, and increased cell survival (295, 297, 300–302).

Strong epidemiological evidence shows that people without GHR (Laron dwarfism) live healthy normal lives despite low IGF-1 levels. Strikingly, they do not develop cancer (nor diabetes), while overabundance of GH/IGF-1 links to cancer incidence (280, 292, 309). For common cancers (breast, colon, prostate, melanoma) tall size relates to cancer risk (308, 310–314). Most importantly, studies with cells, tissues and animals show that GH/IGF-1 stimulates growth of these same cancers, while cancer growth without GH/IGF-1 activity is absent (57, 294, 300, 301, 305). Recent data suggest that also pancreatic ductal adenocarcinomas and small-cell and squamous-cell lung cancer are driven by GH/IGF-1 (21, 315, 316). Thus, there is solid evidence that both GH and IGF-1 are important cancer drivers in humans.

Most cancer cells express GH. This raises the possibility that autocrine GHR activation might be a cancer-driver. In breast cancer tissue, that is devoid of pituitary-specific POU domain transcription factor 1, GH expression is stimulated by progesterone (58, 317). Approximately two-thirds of human breast cancers are steroid hormone receptor (ER/PR) positive and treated with combinations of selective estrogen receptor modulators. It is currently unknown whether their effect relies on inhibition of GH expression. Whether triple-negative breast cancer (TNBC) cells, that do not respond to these modulators, also depend on GH expression, is unknown. The GH-antagonist, pegvisomant, has been successfully used in cancer-derived cell lines (295), but does not inhibit autocrine-acting GH in cancer patients as it probably cannot interfere with intracellular GH/GHR signaling (82). Direct targeting of GH signaling is therefore most probably the only possibility for therapeutic intervention in most cancers.

GH treatment of intestinal organoids closely recapitulating normal human intestinal mucosa resulted in p53 suppression and increased Wnt (318). GH treatment leads to down-regulation of E-cadherin, which controls cell adhesion and prevents tumor cell dissemination (318, 319). An interesting JAK-STAT3 and Wnt–β-catenin pathway connection was revealed that fuels the growth of intestine tumors. The study presents evidence that partial suppression of systemic JAK-STAT3 signaling is sufficient to limit tumor growth by reducing Bmi-1–dependent repression of p21 and p16. Normally p21 is repressed by Bmi-1 in APC-mutant tumors (320, 321). This connection provides a route to use the GHR-STAT3 pathway for a therapeutic modality to inhibit APC mutant cancers.

A growing number of studies implicate GH also in development of therapeutic resistance in a variety of human cancers (322). Both JAK2- and Lyn-initiated pathways activate, upon anti-cancer treatment, many different systems that upregulate ABC-multidrug efflux pumps (ABCG2), block apoptosis, DNA repair (p53), and pro-apoptotic molecules (Bax, PPARγ), suppresses caspase activation, and induce EMT and markers of stemness like ALDH1, NANOG, and CD24. In melanoma, GH upregulates the melanocyte-inducing transcription factor (MITF), that targets the oncogene MET, and organizes the resistance to radiation therapy (323).

## The Regulation of the GHR at the Cellular Level

Given the many factors that control the GHR activity it is important to integrate this knowledge into a concept. Except for autocrine signaling, the number of GHRs at the cell surface determines the sensitivity for GH. This is tightly controlled by the factors discussed above.

Within 20–30 min after synthesis in the ER, the GHR arrives at the cell surface, is available for GH binding during ~30 min, is endocytosed, and degraded within 5 min. A good indication for this can be delineated from the ratio between the precursor form in the endoplasmic reticulum (110,000 Mr) and the mature form at the cell surface (130,000 Mr) if separated by SDS-PAGE and immunoblotted from a crude cell extract using an anti-GHR antibody: If this ratio is approximately 1:1 at steady state, it implies that each GHR is present at the cell surface for only 30 min. Protease K treatment on ice shows that almost no 130,000-Mr species is inside, on route to the lysosomes. In **Figures 2**, **5** the controlling factors are shown.

Based on data from literature combined with our own data we propose the following concept for GHR endocytosis. The major regulators are: ADAM17, JAK2, Ubc13/CHIP/Proteasome, CK2/βTrCP, βTrCP (DSGxxS), and SOCS2. In steady state (no GH), endocytosis is enabled by Ubc13/CHIP and βTrCP (DSGxxS). Both systems are necessary and sufficient. If JAK2 is activated, S341 is phosphorylated, presumably by an activated CK2, SOCS2 binds to pY487/pY595, and all 4 ubiquitination systems are necessary and sufficient for endocytosis. Inactivation of each impedes endocytosis and prolongs GHR signaling capacity. Summarizing the contribution of each enzyme system to GH sensitivity is as follows:

ADAM17 (TACE) contributes ~10% to the inactivation of GHR (**Figure 2**). It is involved in the shedding of many membrane proteins and receptors. Its activation, whether that means increased presence at the cell surface, increased enzymatic activity *via* phosphorylation, or increased residence time at the cell surface is poorly documented. Many signaling pathways are known to promote ADAM17 phosphorylation including PKCs, ERKs, p38 MAPK and PLK2 (324). Shedding of the GH-GHR complex is prohibited (39, 48, 152). Interestingly, Uev1A-Ubc13 (see below) mediates the classical TNFα-induced NF-κB signaling pathway, and at the same time provides for a feedback loop together with CHIP to terminate NF-κB signaling by facilitating ADAM17 maturation *via* RHBDF2 ubiquitination (325). It would not be surprising if GHR signaling would act on ADAM17 in an analogous way.

High JAK2 levels inhibit GHR endocytosis, but in normal cells and tissues this might be not relevant. In IM9 lymphoblasts high levels of JAK2 may prolong the life time at the cell surface (92), but in γ2A cells, that do not express JAK2, GHR has a normal half-life (326). In addition to being controlled by ancillary factors such as SH2-B (91), their activities are subjected to auto-activation, they trans-phosphorylate tyrosine residues in specific patterns (327), they detach from activated receptors to be recycled by phosphatases (92), and in particular, JAK2 responds to heat stress by irreversible aggregation (103). Thus, elevated body temperatures lower the responsiveness of cytokine receptors, and consequently, contribute to a balanced immune system e.g. during a cytokine storm (328, 329).

Ubc13/CHIP/Proteasome are required for both unstimulated and GH-stimulated (pGHR) endocytosis (35). The exact mechanism is still unknown. The bottom-line is that endocytosis of the GHR is not possible if either proteasome activity, CHIP or Ubc13 are lacking. Other observations include: Endocytosis does not require lysine residues, and proteasome-independent endocytosis is only possible if the tail is partly truncated (40, 170). Given its nature as co-chaperone Ubc13/CHIP/Proteasome most likely act in a late step in GHR endocytosis, analogous to TPR proteins in quality control of mislocalized membrane proteins (34, 330). In an analogous way, we propose that CHIP binds with its first TPR motif to the UbE motif, with its central TPR motif to the proteasome and with its U-box to the ubiquitin conjugase Ubc13/UEV1a. Recruited by CHIP, the proteasome might remove the C-terminal part of the GHR downstream of the DSGxxS motif at amino acid 383: an action that explains why endocytosis of a truncated GHR requires both UbE and DSGxxS motifs, but no proteasomal activity (43). This is in line with our observation that degradation of the GHR cytosolic domain precedes degradation of the GH-binding domain (331). In this role CHIP cleans up and definitively terminates GH signaling at the cell surface. In such a scenario, proteasome inhibitors might be considered pro-cancer and pro-aging, as they prolong the residence time of the GHR at the cell surface (39, 209).

Phosphorylation of S341 in the UbE/TPR motif, presumably by CK2, is responsible for an 150% increase of the rate of GHR endocytosis and degradation (179). We showed that this is GH-induced, but also other conditions and stressors might stimulate S341 phosphorylation. Insulin has been suggested to reduce GHR levels and GH signaling in PI-3 kinase- and MAPK-dependent manners (332–334). Also, IGF-1 and estrogen might use phosphorylation of S341 to decrease the pool of GHRs at the cell surface (335, 336). Pro-inflammatory cytokines such as TNFα and IL-6 have been described to induce GH insensitivity. In the latter case, the kinase acts to desensitize cells for GH (337). Coincidentally or not, neighboring JAK2 that binds 45 amino acid residues upstream also is a CK2 client (338). If stressors phosphorylate the S341 in the UbE/TPR motif, the GHR endocytosis rate is increased, independent of SOCS2 (see below).

Phosphorylation of the DSGxxS motif: The DSGRTS sequence in GHR seems to be phosphorylated in basal conditions, without applying any special treatment or stressor to the cells (42). However, it is possible that under physiological conditions the phosphorylation status of DSGRTS might also be regulatable upon certain stimuli, as for the UbE/TPR motif. Additionally, in some situations that require high levels of GH signaling, the body would benefit from higher sensitivity to GH at steady state, e.g. in the process of chondrogenesis at children’s growth plate, or during adolescence to stimulate breast growth, or at the end of mitosis if cells need to growth (339, 340). A decreased kinase activity towards the DSGRTS serine residues would result in decreased basal degradation of GHR, increase in cellular levels of GHR, and consequently increase in GH sensitivity.

SOCSs induce proteasomal degradation of targets through ubiquitination (341, 342). However, in case of transmembrane receptors like GHR and prolactin receptors it is less clear, whether they contribute only to their signal termination or also to their endocytosis/lysosomal targeting/degradation. SOCS2 binds to a degron sequence TP^495^AGS downstream of the STAT5b-interacting pY487. If P495 is mutated to threonine, the binding to SOCS2 is prevented, and the degradation of activated GHR (pGHR) is delayed (21). Assuming that the GHR-JAK2 complex *initiates* signaling only from the cell surface, the results of Chhabra and co-workers imply that pGHR can endocytose and be send to lysosomes, only after SOCS2 has acted on the pGHR (at the cell surface). In that case, SOCS2 is part of a concerted action together with JAK2, βTrCP, and CHIP in initiating signal transduction and preparing the GHR for endocytosis (**Figures 2** and **5**). Increased time span between signal initiation and endocytosis intensifies GH-induced signaling per GHR complex. In a previous study we showed that signal transduction can continue after endocytosis (343). At that time a clear distinction between events starting at the cell surface and continuing inside was difficult to make. Endocytosis of GHR with all tyrosine residues deleted is near normal (92). Thus, SOCS2 contributes only to the deactivation and endocytosis of pY487, pY595-GHR. If the GH/GHR signaling comes from inside (autocrine activation), as is assumed in many cancer cells, there are many open questions: Do the activated GH/GHR complexes reach the cell surface or are they directly transported to MVBs, what are the signaling modes and capacities of these complexes and (how) is it regulated? These are important open questions to understand the role of the GHR in cancer growth.

STAT5b binding to tyrosine residues 487, 534, and 627 are most important for its activation (22). Like GHR knock-out mice, mice that express GHR(1-391) show insulin sensitivity with obesity (344). GH-mediated STAT5b activation acts on multiple sites in the major insulin responsive tissues to promote insulin sensitivity. These actions are regulated at both transcriptional and posttranscriptional levels, and although ChIP analysis indicates direct STAT5b action at the promoter level of key genes, it is apparent that many of the insulin-sensitizing actions of GH-STAT5b deficiency are indirect.

Tyr627: In an effort to determine the contribution of individual tyrosine residues to the STAT5b and MAPK signaling pathways, we found that the Y627F mutation resulted in constitutive GHR, JAK2 and MAPK phosphorylation and activation (22). It is expected that this mutation would act as pro-cancer and pro-aging, but to our knowledge, this variant has not been reported in humans yet (345, 346).

In this summary, the role of Lyn *per se* has not been discussed, as the molecular details as how it functionally interacts with JAK2 and the endocytosis machinery are unknown.

## Conclusions and Perspectives

In this review, we discussed enzyme systems that regulate the GH-sensitivity of cells by direct interaction (ADAM17, JAK2, βTrCP, CHIP, SOCS2). As discussed in the previous section, except for JAK2, delayed activity of each of the systems predicts prolongation of the GHR at the cell surface. As GHR synthesis continues, delayed endocytosis results in increased GH-sensitivity. This is illustrated for SOCS2: prolonged GH signaling, due to a defective GHR-SOCS2 interaction, promotes cancer progression in human lung cancer (21, 202). Although the GH/IGF-1 axis is clearly involved in cancer, none of the other binding sites have yet been identified in genome-wide association study (204, 205). Apparently, these sites are highly relevant for life and do not allow mutations.

**Figure 7:**
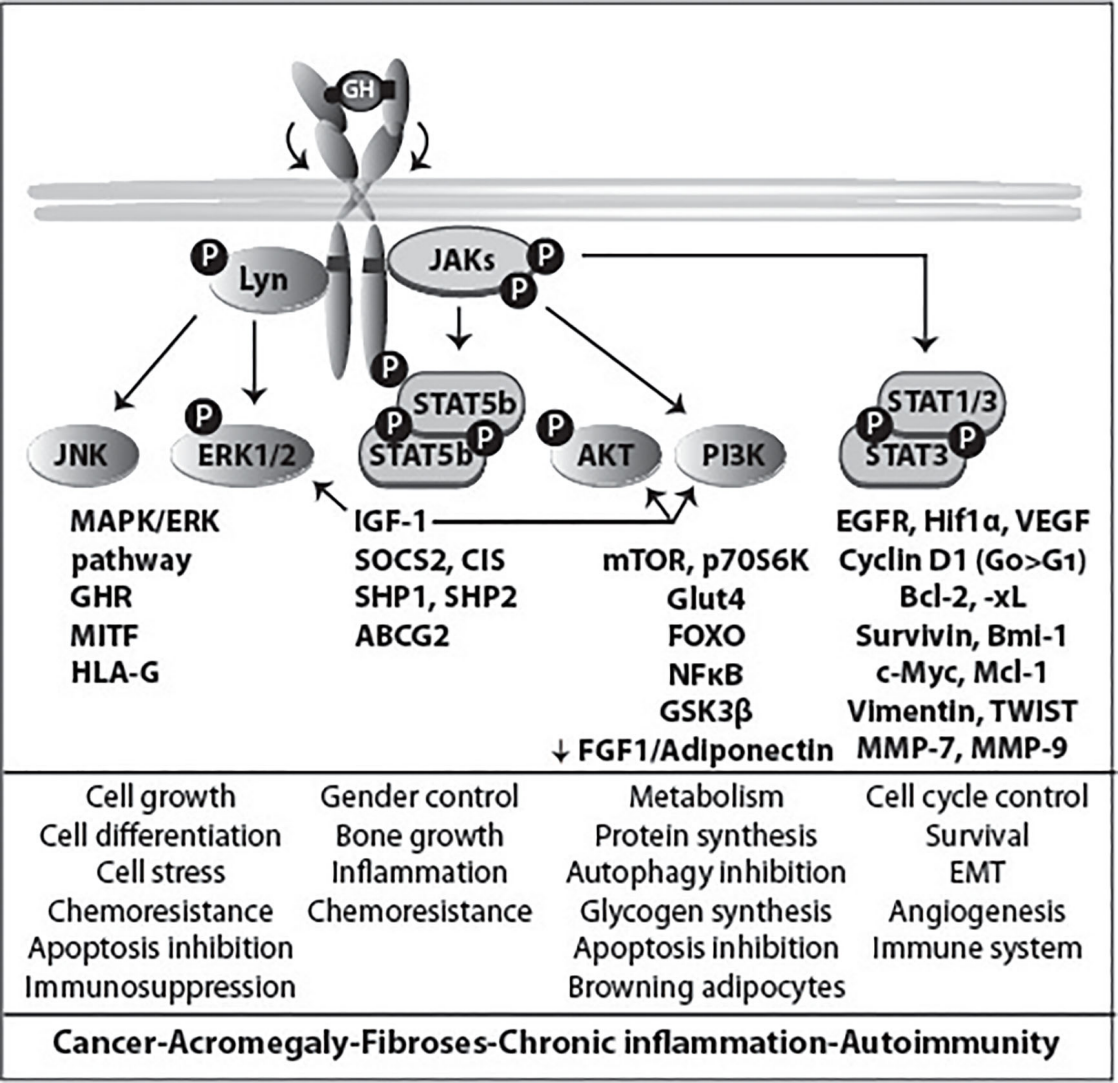
GHR signaling and its span of control. GHR acts *via* JAK/STAT and Lyn, and controls many aspects of cell growth and differentiation, gender, metabolism and cell cycle (middle panel). The general scheme is in **Figure 4**. Deregulated downstream effectors have been studied in great detail in promoting many cancers and chronic diseases (lower panel).

Considering the GH/IGF-1 axis as the mains switch, downstream effectors are the executers of the many tasks (**Figure 7**) (116). Without exception, these factors function in various signaling pathways and are regulated not only by signaling receptors but also in networks of other stressors. Obviously, mutations in these crucial “house-hold factors” contribute to several chronic diseases. However, in many instances, they need the *supervision* of the GH/IGF-1 axis. As each of the enzyme systems impact the activity of the GHR similarly (less interaction results in higher GH-sensitivity) it is plausible that their presence in the regulation of the hierarchical command-and-control mode of the GHR indicates their importance for major control systems. Accordingly, their loss of function would result in the same effect as GHR gain of function. Upregulation of the GH/IGF-1 axis in adult species leads to chronic diseases as illustrated in **Figure 7** (4–10, 12, 13, 347). Indeed, there are many studies that validate this hypothesis. Taken together, literature on CHIP and SOCS shows striking analogy with the regulatory potential of the GHR: organisms with increased CHIP and SOCS2 activity live longer and suffer less from chronic diseases. This is also observed for some of the downstream GHR effectors like mTOR (348). Controlling GHR *turnover* in the axis: GHRH → GHRHR → GH → GHR (+IGF-1) → Growth/Aging, is a challenging mission. Accomplishment will be highly rewarding as it might offer novel tools to fight the conditions, that underlie the major diseases of aging populations.

## Author Contributions

All the authors contributed equally. All authors contributed to the article and approved the submitted version.

## Funding

This work was supported by the European Network of Excellence, RUBICON “Role of ubiquitin and ubiquitin-like modifiers in cellular regulation”, grant LSHG-CT-2005-018683; two European Union Marie Curie Networks, grant MRTNCT-2006-034555 (UbiRegulators) and ERBFMRXCT96-0026; by the Dutch Technology Foundation Stichting Technische Wetenschappen (STW), which is the applied science division of the Nederlandse Organisatie voor Wetenschappelijk Onderzoek (NWO), and the Technology Program of the Ministry of Economic Affairs, Grant 11155: “Targeting the Jak2-GH receptor interaction for treatment of cancer”, The Netherlands Proteomics Centre, “Proteomic analysis of ubiquitylation in membrane trafficking, NPC3.1, and the Netherlands Organization for Scientific Research Grants NWO-ALW 814-02-011 and NWO-902-23-192.

## Conflict of Interest

GS was employed by BIMINI Biotech. B.V., Leiden.

The remaining authors declare that the research was conducted in the absence of any commercial or financial relationships that could be construed as a potential conflict of interest.
